# A UiO-66 3D photonic crystal optical sensor for highly efficient chlorobenzene vapor detection†

**DOI:** 10.1039/d2ra05494a

**Published:** 2022-10-24

**Authors:** Yaru Wang, Zhaolong Wang, Yangfan Gao, Yi Yuan, Jianfei Liu, Jun Yan, Yunlin Chen

**Affiliations:** School of Physical Science and Engineering, Beijing Jiaotong University Beijing 100044 China ylchen@bjtu.edu.cn

## Abstract

Chlorobenzene (C_6_H_5_Cl) is a flammable liquid with high vapor activity, which is a severe threat to the environment and human health. Therefore, it is essential to develop a highly efficient sensor to detect C_6_H_5_Cl vapor. Herein, we developed a UiO-66 three-dimensional photonic crystal (3D PC) optical sensor and investigated its sensing properties toward the C_6_H_5_Cl vapor. The UiO-66 3D PCs optical sensor shows a high sensitivity of C_6_H_5_Cl vapor, in the concentrations range of 0–500 ppm, the reflectance intensity response to be 0.06% ppm with a good linear relationship, detection limit can reach 1.64 ppm and the quality factor is 10.8. Additionally, the UiO-66 3D PC optical sensor demonstrated great selectivity with the values of selectivity (*S*) varying from 2.24 to 10.65 for the C_6_H_5_Cl vapor as compared with carbon tetrachloride (CCl_4_), dichloromethane (CH_2_Cl_2_), 1,1,2-trichloroethane (C_2_H_3_Cl_3_), benzene (C_6_H_6_), deionized water (H_2_O), ethanol (C_2_H_5_OH) and methyl alcohol (CH_3_OH) vapors. Moreover, the UiO-66 3D PC optical sensor shows an ultrafast optical response time and recovery times of 0.5 s and 0.45 s with exceptional stability and repeatability to 500 ppm C_6_H_5_Cl vapor. These excellent sensing properties are attributed to the efficacy of signal transduction, increased porosity and gas adsorption sites, which are intrinsically endowed by the design of the 3D optical structure. The design and fabrication of this UiO-66 3D PC optical sensor might open up potential applications for the detection of the C_6_H_5_Cl vapor.

## Abstract 2

Chlorobenzene (C_6_H_5_Cl) is a flammable liquid with high vapor activity, which is a severe threat to the environment and human health.

## Graphical abstract

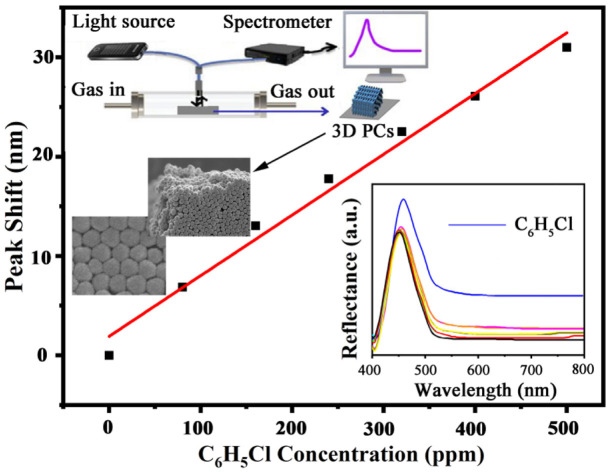

## 1 Introduction

Chlorobenzene (C_6_H_5_Cl), a poisonous and easily explosive gas with a pungent odor, is primarily found in industrial emissions and generated during chemical manufacturing.^1^ The long-term inhalation of chlorobenzene vapor causes headache, nausea and vomiting, affecting the human life and health.^2^ Therefore, it is extremely important to achieve an efficient and advanced detection of C_6_H_5_Cl vapor to reduce environmental and health risks. To date, a number of gas sensors for C_6_H_5_Cl, such as electrochemical gas sensors,^3^ metal oxide semiconductor (MOS) gas sensors^4,5^ and optical sensors,^6^ have been proposed. Among them, optical sensors have received more attention due to: (1) low cost (the gas sensing mechanism is a physical process and not a chemical reaction), (2) convenience and safety, (3) the possibility to select the appropriate wavelength to detect a specific gas and (4) strong anti-electromagnetic interference ability.^7,8^ Optical sensors include localized surface plasmon resonance-based sensors, Fabry–Perot thin films, fluorescent sensors, colorimetric sensors and metal–organic framework (MOF) sensors. The most studied optical sensors in the literature are the MOF sensors.^9,10^

Zirconium-based metal–organic frameworks (Zr-MOFs) are used in sensing applications due to the strong Zr–carboxylate interactions as well as large surface area, tunable pore structure and excellent thermal and chemical stability.^11^ UiO-66 with a face-centered-cubic structure is the archetype of Zr-MOFs.^12^ Photonic crystals (PCs) have gained considerable attention in the field of optical sensors thanks to their unique periodic nano-/microstructures and photonic characteristics.^13,14^ There are three major types of PCs, namely one-dimensional (1D), two-dimensional (2D) and three-dimensional (3D) PCs. Compared with 1D and 2D PCs, 3D PCs are advantageous in optical sensors due to their sensing units with tunable patterns, which result in a complete photonic bandgap (PBG) at visible wavelengths.^15^ They can convert the signals of external stimuli into easily detectable optical signals when employed as sensors. The porosity and stability of UiO-66 combined with the unique periodic structure and efficacy of signal transduction of 3D PCs have greatly facilitated the optimization of device performance.

In recent years, the construction of UiO-66 3D PCs has been extensively studied. Lu *et al.* prepared UiO-66 three-dimensional superlattices by sedimentation self-assembly, which illustrated that the self-assembly technique is a reliable route for fabricating MOF-based functional materials and devices with ordered structures.^16^ Avci *et al.* prepared UiO-66 three-dimensional ordered superstructures by the self-assembly of UiO-66 crystals and found that the superstructures were porous and showed a photonic bandgap functionality.^17^ Recently, Wang *et al.* reported various types of MOF superparticles by the self-assembly of different polyhedral MOF particles (including UiO-66) and explored the shape dependence of the internal structure. They established shape-size-coloration relationships by correlating the internal order with microscopic and macroscopic structural coloration.^18^ Lyu *et al.* showed the assembly of 1D chains, 2D films and quasi-3D colloidal superstructures; they also found that these structures were highly tunable and suitable for use in sensing, optics, and photonics.^19^ Compared with the studies on the 3D structure of UiO-66, only a few studies have been carried out on the application of the UiO-66 3D structure. Cui *et al.* reported that the self-assembly, a 3D film of UiO-66 based Langmuir Blodgett technology, exhibited a significant and selective absorbance wavelength shift in response to chemical vapors, such as water, methanol, and DMF.^20^ Yue *et al.* proposed a UiO-66-coated fiber-optic Michelson Interferometer (MI) sensor for fluoride-ion detection based on the layer-by-layer self-assembly technology. The results showed that the sensor exhibited excellent linear response, high sensitivity and fast response time.^21^ Based on the above studies, the combination of UiO-66 particles into three-dimensional photonic crystals (3D PCs) with an ordered superstructure is conducive to the realization of high-performance optical sensors for detecting the vapors of volatile organic compounds (VOCs).

**Scheme 1:**
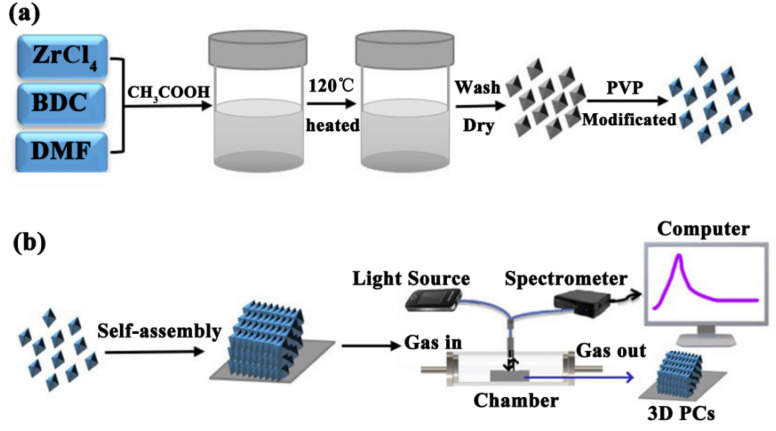
(a) The synthesis of octahedral UiO-66 nanoparticles. (b) The fabrication of the UiO-66 3D PC optical sensor and the experimental setup for sensing tests.

In this study, we prepared a highly efficient UiO-66-based 3D PC optical sensor and investigated its performance in sensing C_6_H_5_Cl vapor. The monodisperse UiO-66 octahedral crystals were synthesized by adding acetic acid to modulate the shape and control the nucleation. The UiO-66 3D PC optical sensor was fabricated based on the assembly of octahedral UiO-66 nanoparticles *via* the solvent evaporation technique for the C_6_H_5_Cl vapor sensing application (Scheme 1). The sensing properties, such as selectivity, sensitivity, response and recovery time, stability and repeatability, of the UiO-66 3D PC optical sensor toward C_6_H_5_Cl vapor were investigated systematically. Furthermore, the C_6_H_5_Cl vapor-sensing mechanism of the UiO-66 3D PC optical sensor is also discussed in detail.

## 2 Experimental methods

### 2.1 Synthesis of UiO-66 nanoparticles

The UiO-66 nanoparticles were synthesized by a solvothermal method (Scheme 1). A mixture of 24.92 mg 1-4-benzendicarboxylic acid (BDC) and 34.95 mg ZrCl_4_ was dissolved in 10 mL of *N*,*N*-dimethylformamide (DMF) containing acetic acid at 0.5, 1.0, 1.5 and 2.0 M, respectively. The mixture was stirred with a magnetic stirrer and reacted at 120 °C for 12 h. After cooling, the colloidal crystals were collected by centrifugation and washed several times with DMF and then methanol. The SEM images of the four samples are provided in Fig. S1.† For modification, the particles were dispersed in 2% PVP (aq.), stirred for 12 hours, and then washed several times with deionized (DI) water. The products collected by centrifugation were redispersed in DI water for self-assembly.

### 2.2 Fabrication of the UiO-66 3D PC optical sensor

The UiO-66 3D PC optical sensor was fabricated by assembling the octahedral UiO-66 nanoparticles *via* the solvent evaporation technique on a silicon substrate. The silicon substrate was washed with ethanol and DI water. 100 μL of the colloidal dispersion of UiO-66 particles was dropped on the clean substrate and dried at room temperature.

### 2.3 Sensing property tests

The sensing performance of the UiO-66 3D PC optical sensor toward VOCs was tested by an optical fiber spectrometer FX2000-RD. Scheme 1(b) shows the experimental equipment. Briefly, the UiO-66 3D PC optical sensor was placed in a chamber (47.5 cm × 32.5 cm × 26 cm) in the darkroom. The incident light was from a broadband white light source, and the incident angle was 90°. The optical fiber spectrometer delivered the incident light to the UiO-66 3D PC optical sensor and collected the original reflected signal. Then, various vapors were allowed to enter the chamber, and the corresponding reflected signals were collected. The UiO-66 3D PC optical sensor was vacuum-activated for 12 hours before every sensing test to ensure data accuracy.

## 3 Results and discussion

### 3.1 Synthesis and characteristics of UiO-66 nanoparticles

**Fig. 1:**
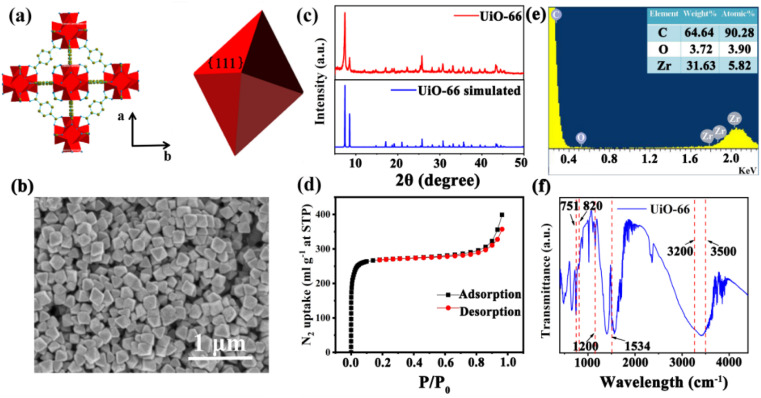
The structure and characterization of the octahedral UiO-66 nanoparticles. (a) The simulated crystal structure of octahedral UiO-66, showing the {111} facets. (b) The SEM image of the octahedral UiO-66 nanoparticles synthesized with 2.0 M acetic acid. (c) The XRD patterns of the synthesized and simulated octahedral UiO-66 nanomaterials. (d) The N_2_ adsorption/desorption isotherm of the octahedral UiO-66 nanoparticles at 77 K and 1 bar. (e) The EDS of the octahedral UiO-66 nanoparticles. (f) The Fourier transform infrared spectrum (FTIR) of the octahedral UiO-66 nanoparticles.

UiO-66 is a Zr-MOF with a face-centered cubic (fcc) structure. As shown in Fig. 1(a), the Zr ion is 8-coordinated by oxygen atoms, and six of these clusters form the Zr_6_O_4_(OH)_4_ metal center; each Zr_6_O_4_(OH)_4_ metal center is connected to 12 nearest neighbor metal centers *via* 12 BDC linkers.^22^ Therefore, UiO-66 exhibits excellent chemical stability and thermal stability, which suit a lot of porosity-related applications, such as gas sensing, catalysis and storage.^23^ The UiO-66 nanoparticles were synthesized by a solvothermal method in this work. In a typical synthesis reaction, a mixture of terephthalic acid (BDC) and ZrCl_4_ was dissolved in *N*,*N*-dimethylformamide (DMF) containing acetic acid, reacted at 120 °C for 12 h,^16^ and acetic acid was added as a modulator to control the shape and nucleation of UiO-66. The shape of the UiO-66 nanoparticles was modulated by changing the concentration of acetic acid in the process from 0.5 M to 2.0 M; the detailed parameters are shown in Table S1.† The SEM images in Fig. S1(a) and (b)† show that when the acetic acid concentration was 0.5–1.0 M, the morphology of the UiO-66 nanoparticles was irregular and not monodisperse, and the particle size is not clear. Fig. S1(c)† shows that the UiO-66 nanoparticles tended to be uniform, and incomplete octahedra were formed with the addition of acetic acid at 1.5 M concentration. It can be observed from Fig. S1(d)† that monodisperse and well-defined octahedral UiO-66 were obtained, and the particle size was about 170 ± 10 nm as the acetic acid concentration reached 2.0 M. The change in morphology occurs because the competitive coordination of zirconium ions with the acetic acid molecules affects the shape and nucleation of UiO-66.

The monodisperse UiO-66 nanoparticles with a well-defined octahedral structure are key to their self-assembly into perfect three-dimensional photonic crystals. Fig. S1(d)† shows that sample 4 could self-assemble into a 3D PC optical sensor. The crystallinity and purity of the octahedral UiO-66 nanoparticles (sample 4) were analyzed by XRD and EDS. Fig. 1(c) shows the XRD pattern of octahedral UiO-66; the (111) plane exhibits the strongest diffraction peak, which reveals the exposure of the (111) plane. The peaks are remarkably consistent with the simulated pattern, confirming the purity and high crystallinity of UiO-66. The EDS pattern in Fig. 1(e) shows that the percentage content of carbon (C) atoms, oxygen (O) atoms, and zirconium (Zr) atoms were 90.28%, 3.90%, and 5.28%, respectively. The absence of other elemental impurities demonstrates the high purity of the octahedral UiO-66 nanoparticles. The porosity of octahedral UiO-66 was investigated using the nitrogen adsorption/desorption isotherm. As shown in Fig. 1(d), octahedral UiO-66 displayed a type I isotherm; the nitrogen uptake increased rapidly at low relative pressure (<0.01), demonstrating a microporous property. The specific surface area of octahedral UiO-66 was about 957.25 and 1199.42 m^2^ g^−1^, as calculated by the Brunauer–Emmett–Teller (BET) and Langmuir methods, respectively. Fig. 1(f) displays the FTIR spectrum of the octahedral UiO-66 nanoparticles. The characteristic peaks located at 751–820 cm^−1^ represent the bending vibration of O–C–O, while the peak at 1200 cm^−1^ denotes the vibration of the benzene ring, and the peak at 1534 cm^−1^ is attributed to the vibration of the C–O bond, whereas the 3500–3200 cm^−1^ peak is that of –OH. These results are in agreement with the results reported in ref. 24 and 25.

### 3.2 Synthesis and characteristics of UiO-66 3D PCs

**Fig. 2:**
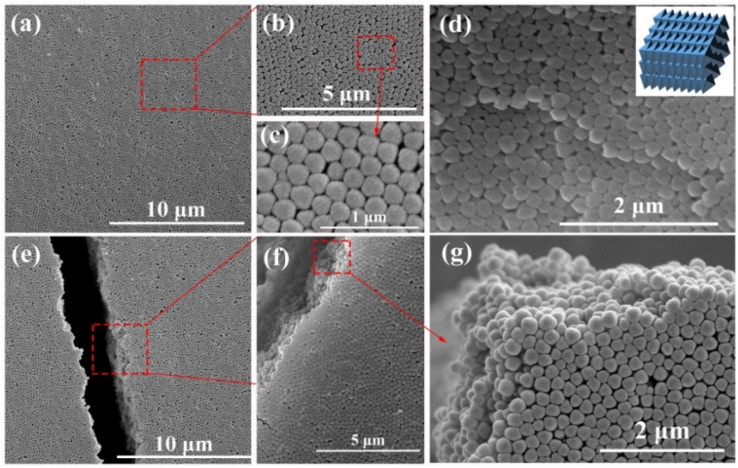
The SEM images of the 3D PCs made using octahedral UiO-66 on a Si substrate. (a) The surface of the large-scale 3D PCs. (b and c) Particle orientation in the magnified image reveals the maintenance of long-range order across the surface. (d) The edge surface of the 3D PCs, showing a terrace structure. Inset: the simulated 3D PCs. (e) The truncated surface of a 3D PC and (f, g) its high-magnification image, showing long-range order in the three dimensions.

The synthetic procedure of UiO-66 3D PCs is illustrated in Scheme 1. The surface of the octahedral UiO-66 nanoparticles was modified with amphiphilic poly(vinylpyrrolidone) (PVP) because non-adsorbed ionic polymers/surfactants in the solution can balance the strong van der Waals (vdW) attraction between the nanoparticles and assist self-assembly by inducing depletion attraction.^26^ Upon self-assembly, the PVP-modified colloidal dispersions of the octahedral UiO-66 nanoparticles spontaneously adopted ordered structures with solvent evaporation. It can be clearly seen in Fig. 2(a)–(c) that dried UiO-66 achieved the densest lattice packing on the surface, and the truncated plane in Fig. 2(e)–(g) further confirm the formation of 3D PCs with long-term order along the three dimensions. In Fig. 2(d), a flat terrace structure with perfect face-to-face contact can be observed when the edge surface of UiO-66 3D PCs at high magnification, and each UiO-66 octahedral nanoparticle is bound to 12 surrounding octahedral nanoparticles by the {111} faces; the inset shows the simulated image of the 3D PCs. The large-area tightly packed arrangement in all the 3D directions will greatly increase the adsorption sites and sensing ability.

### 3.3 Response mechanisms and sensing properties

The microporosity and structure of UiO-66 are highly beneficial for using photonic crystals in sensing applications. The adsorption of vapor in the UiO-66 pore network can change the refractive index, resulting in an apparent shift in the wavelength *λ* of the photonic bandgap spectrum. The diffraction of the 3D PCs can be explained by the Bragg–Snell law, which is demonstrated by the eqn (1):^27^1
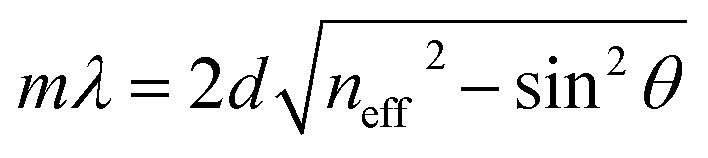
where *m* is the diffraction order, *λ* is the wavelength of reflected light, *d* is the interplanar distance, *n*_eff_ is the mean effective refractive index, and *θ* is the angle of the incident light. The mean effective refractive index *n*_eff_ was calculated using eqn (2):^27^2
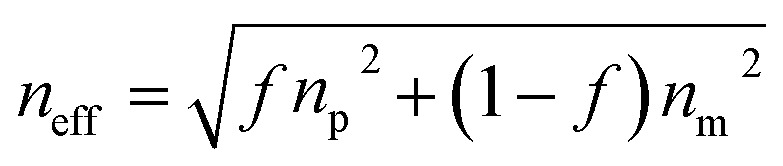
where *f* is the volume fraction of the particles, and *n*_p_ and *n*_m_ are the refractive indexes of the particle and medium, respectively. According to eqn (1) and (2), the reflection wavelength (*λ*) can be tuned by the lattice constant and mean effective refractive index when the incident angle (*θ*) is constant (normal incidence).

**Fig. 3:**
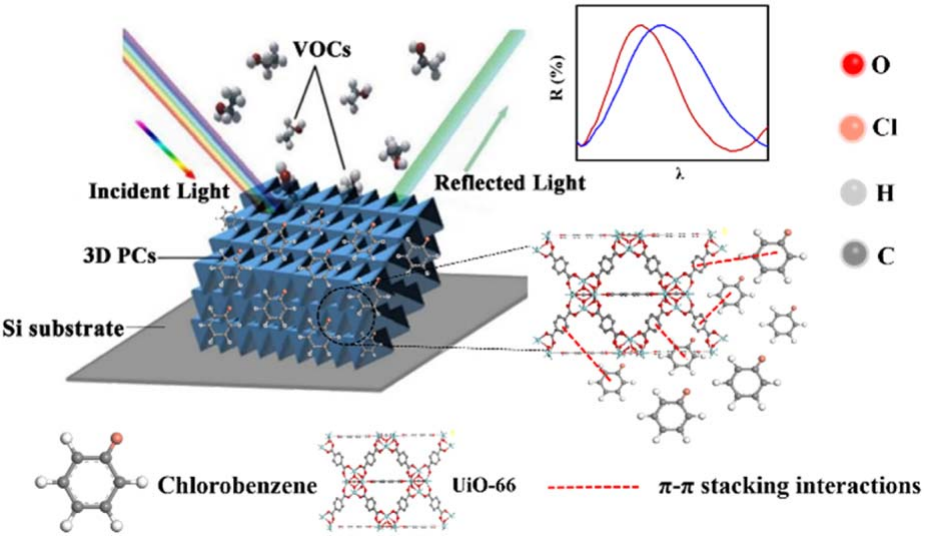
The Schematic of the response mechanism.

Based on the above theory, the response mechanism of the 3D PCs is proposed in this paper to explain the change in the optical signal. The first factor is the refractive index, which can be changed by the penetration ability and concentration of the target substance. The penetrating substances used in this work are the various VOCs (including chlorobenzene). The schematic of the response mechanism is shown in Fig. 3. The second parameter is the lattice constant, which can be tuned by changing particle spacing. The lattice spacing in UiO-66 3D PCs is fixed due to the rigid structure of UiO-66. Therefore, the change in reflection wavelength *λ* is determined by the effective refractive index.

**Fig. 4:**
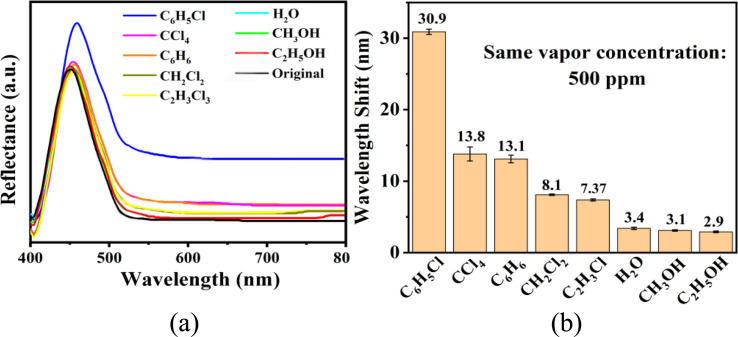
(a) The selectivity of the UiO-66 3D PCs optical sensor to different VOC vapors at 500 ppm. (b) The histogram of the reflected wavelength displacement of the UiO-66 3D PC optical sensor in response to different VOC vapors.

The sensing performance of the UiO-66 3D PC optical sensor was further tested using the reflection spectrum. The indispensable factors for strengthening the performance and practicability of sensors are sensitivity, selectivity, response and recovery time, reusability and stability. Fig. 4(a) reveals the selectivity of the UiO-66 3D PC optical sensor when exposed to H_2_O, C_6_H_5_Cl and various VOC vapors (CCl_4_, C_6_H_6_, CH_2_Cl_2_, C_2_H_3_Cl, CH_3_OH and C_2_H_5_OH) at 500 ppm. Fig. 4(b) shows the wavelength shift of the UiO-66 3D PC optical sensor in response to different analytes in the form of a histogram. The displacements in the presence of the C_6_H_5_Cl, CCl_4_, C_6_H_6_, CH_2_Cl_2_, C_2_H_3_Cl, H_2_O, CH_3_OH and C_2_H_5_OH vapors were by 30.9, 13.8, 13.1, 8.1, 7.37, 3.1 and 2.9 nm, respectively. Obviously, the 3D PC optical sensor showed an evident response to the C_6_H_5_Cl vapor in comparison with the others. The high selectivity toward C_6_H_5_Cl vapor is gained from the octahedral UiO-66 nanoparticles with a symmetric structure, which leads to non-polarity. When non-polar molecules come into contact, they attract each other *via* van der Waals (vdW) force. Thus, the UiO-66 3D PC sensor shows an excellent response toward the non-polar C_6_H_5_Cl molecules. Conversely, the strong polarity of CH_2_Cl_2_, C_2_H_3_Cl, H_2_O, CH_3_OH and C_2_H_5_OH is the reason for the insensitivity of the UiO-66 3D PC optical sensor. Among the non-polar molecules C_6_H_5_Cl, CCl_4_ and C_6_H_6_, the better response to C_6_H_5_Cl can be attributed to the hexatomic ring of carbon atoms, which can form π–π stacking interactions between benzene rings, enhancing the absorption ability further. However, it is worth noting the difference in response to C_6_H_5_Cl vapor and C_6_H_6_ vapor. One possible reason for this is that the overlapping accumulation of the benzene rings and hexatomic rings greatly increases molecular interactions, resulting in enhanced polarity.^25^ As for C_6_H_5_Cl, due to the presence of Cl, the overlapping accumulation would not happen. As a result, the UiO-66 3D PC optical sensor presents a better selectivity to C_6_H_5_Cl vapor.

**Fig. 5:**
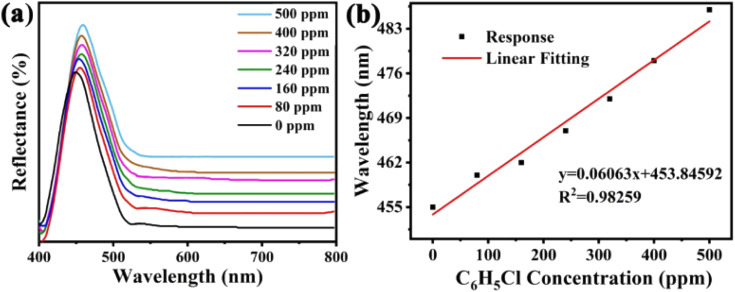
(a) The reflected spectra of the UiO-66 3D PC optical sensor in the 0–500 ppm concentration range of C_6_H_5_Cl vapor. (b) The linear fitting of the wavelengths and C_6_H_5_Cl concentrations detected by the UiO-66 3D PCs optical sensor.

The sensitivity of UiO-66 3D PCs to C_6_H_5_Cl was investigated due to the remarkable distinction in optical signal between C_6_H_5_Cl and other VOC vapors. As shown in Fig. 5(a), the reflection spectrum of the UiO-66 3D PC optical sensor showed obvious displacement when the C_6_H_5_Cl concentration was increased from 0 to 500 ppm. More specifically, when the C_6_H_5_Cl concentration was increased from 80 to 500 ppm (80, 160, 240, 320, 400, 500 ppm), the reflection wavelength shifts were by about 6.88, 13.04, 17.78, 22.53, 26.08, and 30.9 nm, respectively. Fig. 5(b) illustrates a good linear relationship between the wavelength (*λ*) and chlorobenzene concentration. The relationship satisfied the function: *y* = 0.06063*x* + 453.84592, while the slope and coefficient of determination (*R*^2^) were 0.06063 and 0.98259, respectively. The concentration sensitivity (*S*) of the UiO-66 3D PC optical sensor toward C_6_H_5_Cl was 0.06 nm ppm, and the limit of detection (LOD) of C_6_H_5_Cl was calculated by the following equations:^28^3
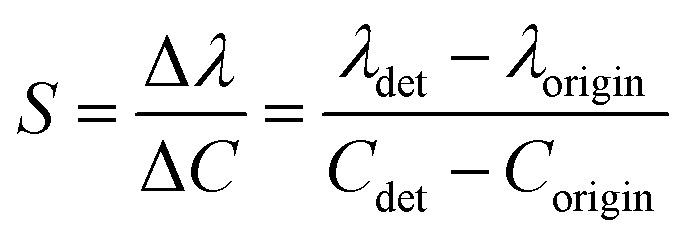
4
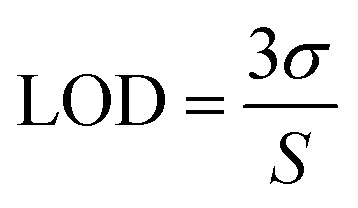
5
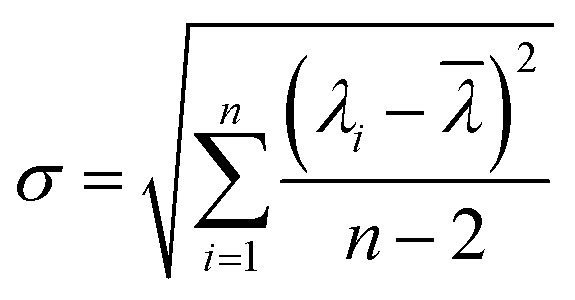
where *S* is the sensitivity, and *σ* is the standard deviation, *λ*_origin_ and *λ*_det_ are the original and detection wavelengths, *C*_origin_ and *C*_det_ are the original and detected concentrations, respectively. At the spectral resolution of 0.1 nm,^28^ the LOD of the UiO-66 3D PC optical sensor toward C_6_H_5_Cl was 1.64 ppm. Additionally, the full width at half maximum (FWHM) was 42.4 nm, while the quality factor (*Q*) and figure of merit (FOM) were respectively calculated to be 10.8 and 1.42 × 10^−3^ ppm by using the following equations:^28^6
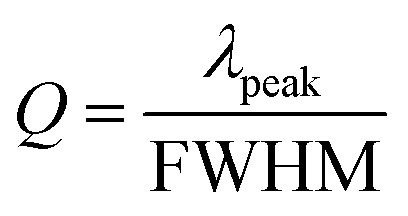
7
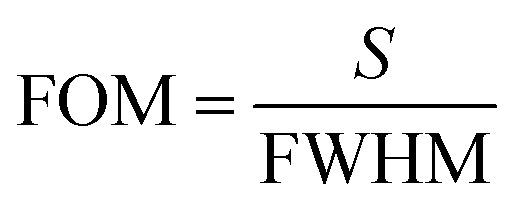
where *λ*_peak_ is the pack wavelength.

**Fig. 6:**
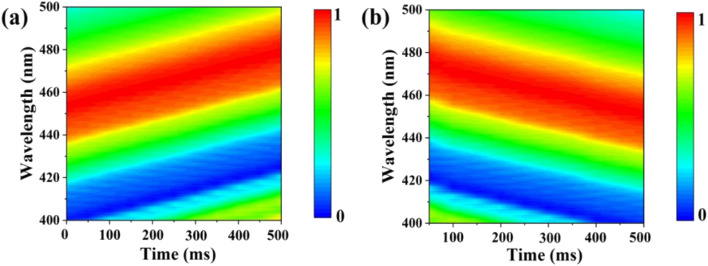
Contour plot variation in the (a) response time of the UiO-66 3D PC optical sensor at 500 ppm chlorobenzene vapor and (b) recovery time of the UiO-66 3D PC optical sensor at 500 ppm chlorobenzene vapor.

The response and recovery times are critical to evaluate the practical application of sensors. Fig. 6(a) and (b) illustrate the response time and recovery time of the UiO-66 3D PC optical sensor. The UiO-66 3D PC optical sensor showed an ultrafast optical response time and recovery time of 0.5 s and 0.45 s at 500 ppm chlorobenzene vapor, respectively, based on the contour plot variation. The key to achieving ultrafast optical response and recovery time is the construction of the UiO-66 3D PCs. UiO-66 owns rapid osmosing channels because of the large specific surface area, a pore-abundant network and excellent structural stability. 3D PCs exhibit enhanced diffusion and a large number of absorption sites for gas molecules in the packed structure in all three dimensions. Moreover, 3D PCs with a complete photonic bandgap (PBG) at visible wavelengths also greatly improve the efficiency of optical signal conversion. Therefore, the UiO-66 3D PC optical sensor demonstrates ultrafast response time and recovery time toward C_6_H_5_Cl vapor. To certify the sensing ability of the UiO-66 3D PC optical sensor, the LOD, response time, and recovery time of the UiO-66 3D PCs optical sensor and other sensors (including UiO-66 1D PCs, UiO-66 3D film sensor for chemical vapors and ZIF-8 1D PCs sensors for chlorobenzene vapor) were compared. As shown in Table 1, the UiO-66 3D PC optical sensor exhibited an ultrafast response time of 0.5 s and recovery time of 0.45 s and high sensitivity of 0.06 nm ppm to chlorobenzene vapor. This proves its better performance and that it is competitive among other optical sensors.

**Table 1:** Performance of MOF-based optical sensor

Optical sensor	Analyte	Limit of detection (LOD)	Response time	Recovery time	Ref.
UiO-66 1D film optical sensor	Ethanol		2 s	3 s	29
UiO-66 2D film optical sensor	Methanol		4 s	25 s	30
UiO-66 2D film optical sensor	Nitrogen		4 s	13 s	30
UiO-66 3D film optical sensor	Chemical vapor		Fast	Fast	21
UiO-66 1D photonic crystal sensor	Hydrochloric acid	0.93 nm ppm	1 s	8 s	31
ZIF-8 1D photonic crystal sensor	Chlorobenzene	6.92 nm ppm	0.6 s	9.5 s	28
UiO-66 1D photonic crystal sensor	Chlorobenzene	13 nm ppm	0.8 s	5 s	25
Porous ZnO nanoplate sensor	Chlorobenzene		5 s	4 s	32
5% Pd@ZnO sensor	Chlorobenzene		19 s	7 s	32
In_2_O_3_ sensors	Chlorobenzene		6.7 s	25.8 s	33
TCN(ii)/KOH/Ni-foam electrochemical gas sensor	Chlorobenzene	1 nm ppm	250 s		3
SmFeO_3_ semiconductive gas sensor	Chlorobenzene		0.5 s	3 s	34
Pt-decorated PSC ZnO NSs sensor	Chlorobenzene		20 s	10 s	5
UiO-66 3D photonic crystals sensor	Chlorobenzene	1.64 nm ppm	0.5 s	0.45 s	This work

**Fig. 7:**
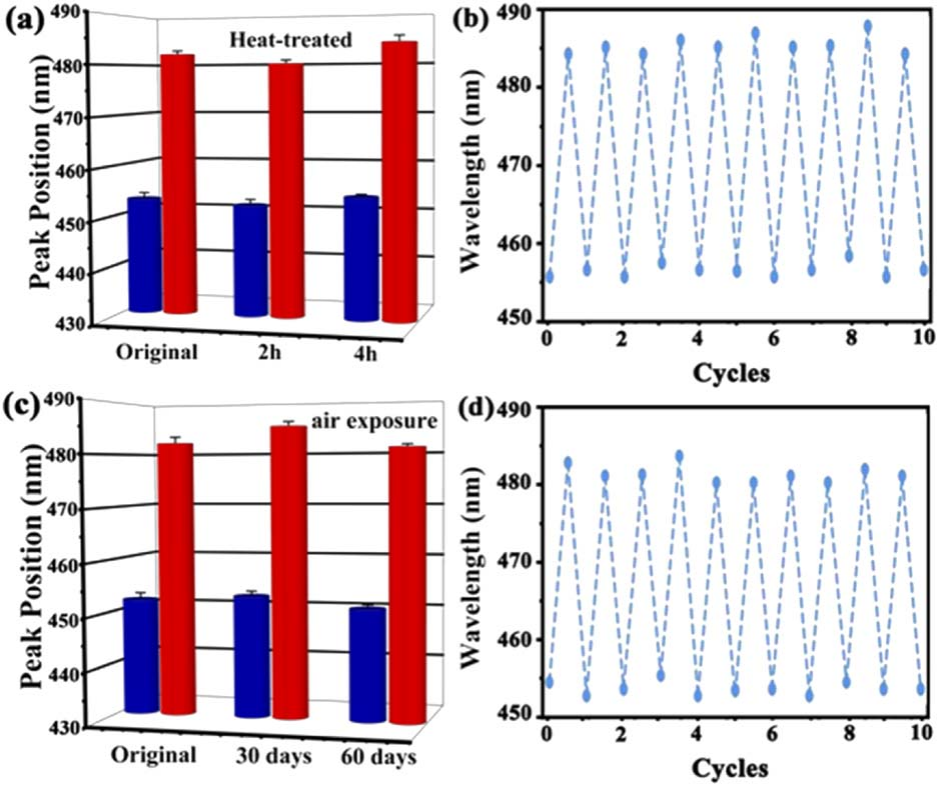
(a) The reflection spectrum of the UiO-66 3D PC optical sensor before and after 2 hours and 4 hours of heat treatment (red: no vapor; blue: 500 ppm chlorobenzene vapor). (b) The repeatability of the UiO-66 3D PC optical sensor after heat treatment (tested at 500 ppm chlorobenzene vapor). (c) The reflection spectrum of the UiO-66 3D PC optical sensor before and after 30 days and 60 days of air exposure (red: no vapor; blue: 500 ppm chlorobenzene vapor). (d) The repeatability of the UiO-66 3D PC optical sensor after air exposure (tested at 500 ppm chlorobenzene vapor).

Stability and repeatability are essential for the long-term application of the UiO-66 3D PC optical sensor. The stability of the UiO-66 3D PC optical sensor was evaluated by measuring the peak positions in the reflection spectrum at 500 ppm C_6_H_5_Cl vapor after two kinds of treatments. Fig. 7(a) indicates the spectrum of the heat-treated UiO-66 3D PC optical sensor at 200 °C for 2 hours and 4 hours. The red column represents the reflection spectrum with no vapor, while the blue column is the reflection spectrum with 500 ppm chlorobenzene after the treatment. Compared with the unheated sensor, the response of the optical sensor to C_6_H_5_Cl vapor was positive after heat treatment for 2 hours and 4 hours at 200 °C. Fig. 7(c) shows the results of the UiO-66 3D PC optical sensor subjected to air exposure for 30 and 60 days; there is no obvious peak shift in the reflection spectrum of C_6_H_5_Cl vapor detection. This affirms the superior thermal stability and long-term storage stability of the UiO-66 3D PC optical sensor. The close framework connections of the Zr–O metal centers to organic linkers result in a highly packed fcc structure, which exhibits unprecedented stability. When the UiO-66 3D PCs are self-assembled from the UiO-66 octahedral crystals *via* the solvent evaporation technique, a dense, fully packed three-dimensional ordered superstructure is formed spontaneously driven by capillary force and the slowly increasing pressure. Therefore, the UiO-66 3D PC optical sensor also owns outstanding stability. Additionally, the repeatability of the UiO-66 3D PC optical sensor was investigated by measuring the variation in the reflection spectrum under 500 ppm C_6_H_5_Cl vapor. Fig. 7(b) and (d) reveal outstanding repeatability in at least ten cycles after heat treatment and air exposure, respectively, and the errors in the refection peak shifts are within 3 nm. The gas sensing process is ascribed to the rapid and reversible physical adsorption of the analytes on the UiO-66 3D PCs. The domination of physical adsorption and desorption do not affect the internal structure, porosity and 3D photonic properties of the UiO-66 3D PCs. Therefore, the UiO-66 3D PC optical sensor exhibits distinguished repeatability.

## 4 Conclusions

In summary, we have demonstrated the fabrication of a UiO-66 3D PC optical sensor based on the assembly of octahedral UiO-66 nanoparticles *via* the solvent evaporation technique. The UiO-66 3D PC optical sensor was designed to highlight the advantages of the porosity and stability of UiO-66 combined with the unique periodic structure and the efficacy of signal transduction of 3D PCs, which greatly promotes the optimization of device performance. The systematic investigation indicates that the UiO-66 3D PC optical sensor displays high sensitivity (about 30.9 nm reflectance peak shift) with a low detection limit (1.64 ppm) and a good quality factor of 10.8 at 0–500 ppm chlorobenzene vapor. The UiO-66 3D PC optical sensor shows outstanding selectivity, with the value of selectivity (*S*) for C_6_H_5_Cl varying from 2.24 to 10.65 compared with those for carbon tetrachloride (CCl_4_), dichloromethane (CH_2_Cl_2_), 1,1,2-trichloroethane (C_2_H_3_Cl_3_), benzene (C_6_H_6_), deionized water (H_2_O), ethanol (C_2_H_5_OH) and methyl alcohol (CH_3_OH) vapors. The extremely high selectivity for chlorobenzene vapor is attributed to the attraction of the van der Waals forces between the nonpolar molecules and the π–π stacking interaction between the benzene rings. Compared with C_6_H_6_, the overlapping accumulation between the benzene rings does not appear in C_6_H_5_Cl because of the Cl group, so the polarity is weakened. Additionally, the results illustrate an ultrafast optical response time (0.5 s) and recovery time (0.45 s) with exceptional repeatability and stability at 500 ppm chlorobenzene vapor. As vapor molecules are generally physisorbed on UiO-66 3D PCs, the sensor is highly reversible and suitable for long-term use. The UiO-66 3D PC optical sensor is expected to be a highly efficient chlorobenzene sensor for environmental monitoring.

## Conflicts of interest

There are no conflicts to declare.

## Supplementary Material

RA-012-D2RA05494A-s001
