# Supplementary material for: A UiO-66 3D photonic crystal optical sensor for highly efficient chlorobenzene vapor detection
Source: RSC Adv. 2022 Oct 24;12(47):30262–9. doi: 10.1039/d2ra05494a (PMC9590246; doi:10.1039/d2ra05494a)
Supplement: RA-012-D2RA05494A-s001 [file RA-012-D2RA05494A-s001.pdf]

Electronic Supplementary Material (ESI) for RSC Advances.
This journal is © The Royal Society of Chemistry 2022

Supplementary information

UiO-66 3D Photonic Crystals Optical Sensor for Highly Efficient Chlorobenzene Vapor Detection

*Yaru Wang, Zhaolong Wang, Yangfan Gao, Yi Yuan, Jianfei Liu, Jun Yan, Yunlin Chen**

School of Physical Science and Engineering, Beijing Jiaotong University, Beijing 100044, China

Corresponding Author

[*] E-mail address: ylchen@bjtu.edu.cn (Y. Chen).

1.General information

Marerials. Zirconium tetrachloride ($ZrCl_4$) was purchased from Shanghai Macklin Biochemical Co., Ltd. Terephthalic acid (BDC, $C_8H_6O_4$, AR, ≥99.5%), 1,1,2-trchloroethane ($C_2H_3Cl_3$), chlorobenzene (C_6H_5Cl) and benzene (C_6H_6) were obtained from Sinopharm Chemical Reagent Co., Ltd. N, N-dimethylformamide (DMF, AR, ≥99.5%), acetic acid (CH_3COOH, AR, ≥99.5%) and carbon tetrachloride (CCl_4, AR, ≥99.5%) were acquired from Tian jin Fuyu Fine Chemical Co., Ltd. Polyvinylpyrrolidone (PVP, Mr=10,000) was purchased from Sinopharm Chemical Reagent Co., Ltd (Shanghai, China). Methyl alcohol (CH_3OH, AR, ≥99.5%), ethanol (CH_3CH_2OH, AR, ≥99.5%) and dichloromethane (CH_2Cl_2, AR, ≥99.5%) were got from Beijing Chemical Works.

Characterization. The surface and cross-section morphologies analysis of 3D PCs were investigated by Scanning Electron Microscope and Energy dispersive X-ray spectroscopy (SEM, EDS, JEM-6700 F). Powder X-ray diffraction (PXRD, Rigaku D/max, λ=1.5418 Å) and Fourier transform infrared spectroscopy (FTIR, SPECTRUM 400) were used to research the performances of UiO-66 powders. The specific surface area was obtained from the N_2 adsorption-desorption isotherms of the sample measured at 77 K using a JW-BK122W system.

2. Supplementary data

Table S1. Experimental parameters.

Sample	$ZrCl_4$ (mg)	BDC (mg)	DMF (ml)	CH_3COOH(M)	Reaction conditions
1	34.9	24.9	10	0.5	120℃ 24h
2	34.9	24.9	10	1.0	120℃ 24h
3	34.9	24.9	10	1.5	120℃ 24h
4	34.9	24.9	10	2.0	120℃ 24h

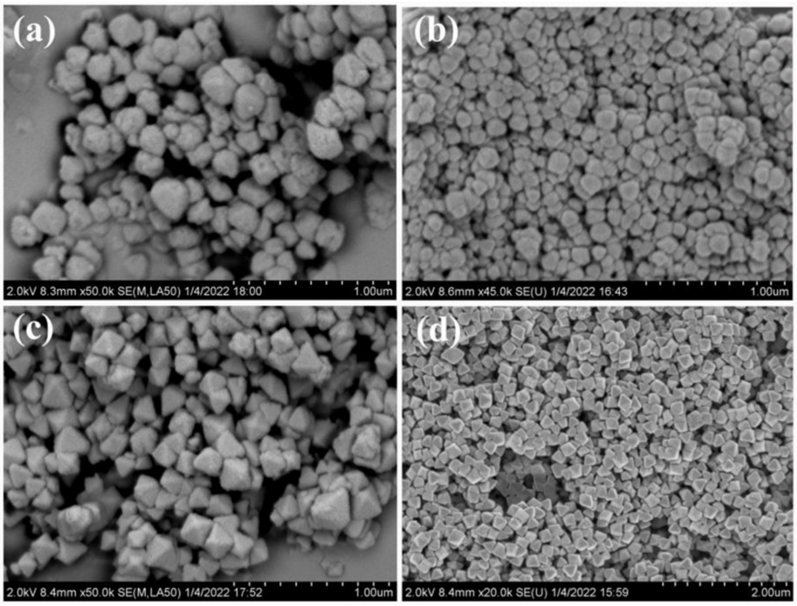

Figure S1. SEM images of UiO-66 nanoparticals synthesized in the presence of (a) 0.5 M acetic acid (sample 1), (b) 1.0 M acetic acid (sample 2), (c) 1.5 M acetic acid (sample 3), (d) 2.0 M acetic acid (sample 4).
